# Quantification of Beat-To-Beat Variability of Action Potential Durations in Langendorff-Perfused Mouse Hearts

**DOI:** 10.3389/fphys.2018.01578

**Published:** 2018-11-27

**Authors:** Gary Tse, Yimei Du, Guoliang Hao, Ka Hou Christien Li, Fiona Yin Wah Chan, Tong Liu, Guangping Li, George Bazoukis, Konstantinos P. Letsas, William K. K. Wu, Shuk Han Cheng, Wing Tak Wong

**Affiliations:** ^1^Department of Medicine and Therapeutics, Faculty of Medicine, Chinese University of Hong Kong, Hong Kong, China; ^2^Li Ka Shing Institute of Health Sciences, Faculty of Medicine, Chinese University of Hong Kong, Hong Kong, China; ^3^Shenzhen Research Institute, The Chinese University of Hong Kong, Shenzhen, China; ^4^Research Center of Ion Channelopathy, Institute of Cardiology, Union Hospital, Tongji Medical College, Huazhong University of Science and Technology, Wuhan, China; ^5^Department of Physiology, Anatomy and Genetics, University of Oxford, Oxford, United Kingdom; ^6^Faculty of Medicine, Newcastle University, Newcastle, United Kingdom; ^7^School of Biological Sciences, University of Cambridge, Cambridge, United Kingdom; ^8^Tianjin Key Laboratory of Ionic-Molecular Function of Cardiovascular Disease, Department of Cardiology, Tianjin Institute of Cardiology, Second Hospital of Tianjin Medical University, Tianjin, China; ^9^Laboratory of Cardiac Electrophysiology, Second Department of Cardiology, Evangelismos General Hospital of Athens, Athens, Greece; ^10^State Key Laboratory of Digestive Disease, Department of anesthesia and Intensive Care, LKS Institute of Health Sciences, The Chinese University of Hong Kong, Hong Kong, China; ^11^Department of Biomedical Sciences, College of Veterinary Medicine and Life Science, City University of Hong Kong, Hong Kong, China; ^12^State Key Laboratory of Marine Pollution at City University of Hong Kong, Hong Kong, China; ^13^Department of Materials Science and Engineering, College of Science and Engineering, City University of Hong Kong, Hong Kong, China; ^14^State Key Laboratory of Agrobiotechnology, School of Life Sciences, Chinese University of Hong Kong, Hong Kong, China

**Keywords:** variability, repolarization, time, frequency, non-linear, entropy

## Abstract

**Background:** Beat-to-beat variability in action potential duration (APD) is an intrinsic property of cardiac tissue and is altered in pro-arrhythmic states. However, it has never been examined in mice.

**Methods:** Left atrial or ventricular monophasic action potentials (MAPs) were recorded from Langendorff-perfused mouse hearts during regular 8 Hz pacing. Time-domain, frequency-domain and non-linear analyses were used to quantify APD variability.

**Results:** Mean atrial APD (90% repolarization) was 23.5 ± 6.3 ms and standard deviation (SD) was 0.9 ± 0.5 ms (*n* = 6 hearts). Coefficient of variation (CoV) was 4.0 ± 1.9% and root mean square (RMS) of successive differences in APDs was 0.3 ± 0.2 ms. The peaks for low- and high-frequency were 0.7 ± 0.5 and 2.7 ± 0.9 Hz, respectively, with percentage powers of 39.0 ± 20.5 and 59.3 ± 22.9%. Poincaré plots of APD_n+1_ against APD_n_ revealed ellipsoid shapes. The ratio of the SD along the line-of-identity (SD2) to the SD perpendicular to the line-of-identity (SD1) was 8.28 ± 4.78. Approximate and sample entropy were 0.57 ± 0.12 and 0.57 ± 0.15, respectively. Detrended fluctuation analysis revealed short- and long-term fluctuation slopes of 1.80 ± 0.15 and 0.85 ± 0.29, respectively. When compared to atrial APDs, ventricular APDs were longer (ANOVA, *P* < 0.05), showed lower mean SD and CoV but similar RMS of successive differences in APDs and showed lower SD2 (*P* < 0.05). No difference in the remaining parameters was observed.

**Conclusion:** Beat-to-beat variability in APD is observed in mouse hearts during regular pacing. Atrial MAPs showed greater degree of variability than ventricular MAPs. Non-linear techniques offer further insights on short-term and long-term variability and signal complexity.

## Introduction

Beat-to-beat variations in the repolarization time-course represent an intrinsic property of cardiac electrophysiological function. This may be manifested as variability of action potential durations (APDs) at the cellular level (Nanasi et al., 2017), or of QT durations at the organism level (Niemeijer et al., 2014; Phadumdeo and Weinberg, 2018). This variability may be affected by distinct physiological states, such as the degree of intercellular coupling (Zaniboni et al., 2000), redox states (Kistamas et al., 2015a), altered intracellular calcium handling (Kistamas et al., 2015b) or APD itself (Abi-Gerges et al., 2010). Clinical studies have shown that higher variability in QT intervals can predict pro-arrhythmic outcomes in the context of non-ischemic heart failure (Hinterseer et al., 2010), as well as long QT syndrome (Hinterseer et al., 2009).

Mouse models are widely used to study cardiac electrophysiological and arrhythmogenic properties, owing to their amenability to pharmacological or genetic manipulation (Nerbonne, 2014; Choy et al., 2016). However, despite the importance of APD variability, it has never been examined in this species. In this study, we quantified beat-to-beat variability in APDs by applying time-domain and non-linear techniques for the first time to monophasic action potential recordings (MAPs) obtained from Langendorff-perfused mouse hearts during regular pacing.

## Materials and methods

### Solutions

Krebs-Henseleit solution (composition in mM: NaCl 119, NaHCO_3_ 25, KCl 4, KH_2_PO_4_ 1.2, MgCl_2_ 1, CaCl_2_ 1.8, glucose 10 and sodium pyruvate 2, pH 7.4), which has been bicarbonate-buffered and bubbled with 95% O_2_-5% CO_2_, was used in the experiments described in this study.

### Preparation of langendorff-perfused mouse hearts

This study was approved by the Animal Welfare and Ethical Review Body at the University of Cambridge. Wild-type mice of 129 genetic background between 5 and 7 months of age were used. They were maintained at room temperature (21 ± 1°C) and were subjected to a 12:12 h light/dark cycle with free access to sterile rodent chow and water in an animal facility. Mice were terminated by dislocation of the cervical spine in accordance with Sections 1(c) and 2 of Schedule 1 of the UK Animals (Scientific Procedures) Act 1986. The technique for Langendorff perfusion has been used by our group and described previously (Tse et al., 2016a,d, 2017). After removal from their chest cavities, the hearts were submerged in ice-cold Krebs-Henseleit solution. The aortas were cannulated using a custom-made 21-gauge cannula prefilled with ice-cold buffer. A micro-aneurysm clip (Harvard Apparatus, UK) was used to secure the hearts onto the Langendorff perfusion system. Retrograde perfusion was carried out at a flow rate of 2 to 2.5 ml min^−1^ by use of a peristaltic pump (Watson–Marlow Bredel pumps model 505S, Falmouth, Cornwall, UK). The perfusate passed through successively 200 and 5 μm filters and warmed to 37°C using a water jacket and circulator before arriving at the aorta. Approximately 90% of the hearts regained their pink color and spontaneous rhythmic activity. These were therefore studied further. The remaining 10% did not and were discarded. The hearts were perfused for a further 20 min to minimize residual effects of endogenous catecholamine release, before their electrophysiology properties were characterized.

### Stimulating procedures

Paired platinum electrodes (1 mm interpole distance) were used to stimulate the right ventricular epicardium electrically. This took place at 8 Hz, using square wave pulses of 2 ms in duration, with a stimulation voltage set to three times the diastolic threshold (Grass S48 Stimulator, Grass-Telefactor, Slough, UK) immediately after the start of perfusion.

### Atrial and ventricular map recording procedures

For atrial MAP recordings, the atrio-ventricular nodes of the Langendorff perfused hearts were first mechanically ablated as previously described (Tse et al., 2016b). This eliminated ventricular far-field activity at the recording electrode. The MAP electrode was placed at the left atrial or ventricular epicardium (Linton Instruments, Harvard Apparatus). The stimulating and recording electrodes were maintained at constant positions separated approximately by a distance of 3 mm. All recordings were performed using a baseline cycle length (BCL) of 125 ms (8 Hz) to exclude rate-dependent differences in action potential durations (APDs). MAPs were pre-amplified using a NL100AK head stage, amplified with a NL 104A amplifier and band pass filtered between 0.5 Hz and 1 kHz using a NL125/6 filter (Neurolog, Hertfordshire, UK) and then digitized (1401plus MKII, Cambridge Electronic Design, Cambridge, UK) at 5 kHz. Waveforms were analyzed using Spike2 software (Cambridge Electronic Design, UK). MAP waveforms that did not match established criteria for MAP signals were rejected (Knollmann et al., 2001; Tse et al., 2016c). They must have stable baselines, fast upstrokes, with no inflections or negative spikes, and a rapid first phase of repolarization. Zero Percent repolarization was measured at the peak of the MAP and Hundred Percent repolarization was measured at the point of return of the potential to baseline (Gussak et al., 2000; Knollmann et al., 2001; Fabritz et al., 2003).

### APD variability analysis

APD variability analysis was performed using Kubios HRV Standard software (Version 3.0.2) over a 60 s period. Time-domain analysis yielded the (1) standard deviation (SD) of APDs, which represents the overall (short-term and long-term) variability, and (2) root mean square (RMSSD) of successive differences of APDs, which represents the short-term variability:

(1) $$\text{SDAPD}=\sqrt{\frac{1}{N-1}\sum_{j=1}^{N}{\left({\text{APD}}_{j}-\text{ A}\bar{\text{PD}}\right)}^{2}}$$

(2) $$\text{RMSSD}=\sqrt{\frac{1}{N-1}\sum_{j=1}^{N-1}{\left({\text{APD}}_{j+1}-{\text{APD}}_{j}\right)}^{2}}$$

Frequency-domain analysis was conducted using the Fast Fourier Transform method. For frequency domain parameters, spectral analysis was performed by using fast-Fourier transform method. The sampling frequency was set to 8 Hz. The power in the repolarization spectrum between 0.04 and 4 Hz was defined as total power (TP). The power in the repolarization spectrum was divided into three different frequency bands: very low frequency power (VLF, 0 to 0.04 Hz), low frequency power (LF, 0.04 to 1.5 Hz) and high frequency power (HF, 1.5 to 4 Hz).

The above frequency analysis does not provide any information on the time evolution of the frequencies. To achieve, this, time-frequency analysis was conducted using two different techniques. Firstly, short-time Fourier transform (STFT) was used to break the signal into small time segments using an appropriate sliding-window function, and then apply a Fourier transformation to the successive sliding-window segments. The Hanning window with a Fast Fourier Transform length of 256 and overlap of 128 were selected.

Secondly, continuous wavelet transform (CWT) was used to divide a continuous-time function into wavelets given by:

(3) $$\text{CWT}(\text{a},\text{b})=\frac{\text{1}}{\sqrt{\text{a}}}\;\int_{-\;\infty }^{\text{+}\infty }\text{x}\left(\text{t}\right)\cdot \psi^{*}(\frac{\text{t}-\text{b}}{\text{a}})\text{dt}$$

Where the superscript, ^*^, is the complex conjugate and ψ_a,b_^*^ represents a translated and scaled complex conjugated mother wavelet. The mother wavelet ψ is invertible when it verifies the condition of admissibility which is stated as:

(4) $$\int_{-\infty }^{\text{+}\infty }\frac{\left|\hat{\psi }(\omega )\right|}{\omega }\text{ d}\omega <\infty$$

The Morlet wavelet was selected, which uses a Gaussian-modulated sinusoid:

(5) $$\psi \left(t\right)=\;\frac{1}{\sqrt[{4}]{\pi }}\left({\text{e}}^{\text{i}\omega_{o}t}-\;{\text{e}}^{-\frac{\omega_{o}^{2}}{2}}\right)\;{\text{e}}^{-\frac{t^{2}}{2}}$$

where ω_o_ is the central frequency of the mother wavelet. The second term in the brackets corrects for the non-zero mean of the complex sinusoid of the first term. This becomes negligible for values of ω_o_ > 5, which we selected in our case:

(6) $$\psi \left(t\right)=\;\frac{1}{\sqrt[{4}]{\pi }}{\text{e}}^{\text{i}\omega_{o}t}\;{\text{e}}^{-\frac{t^{2}}{2}}$$

Non-linear properties of APD variability were studied as follow. Poincaré plots are graphical representations of the correlation between successive APD values, in which APD_n+1_ is plotted against APD_n_. This enables determination of the SD of the points perpendicular to the line-of-identity (SD1). Different points along this perpendicular axis represent a beat-to-beat variation between the initial (*n*) and subsequent (*n* + 1) contraction, representing multiple two-beat “snapshots” with little correlation to a progressive time parameter. Therefore, SD1 is associated with instantaneous or short-term variability. As for the points along the line-of-identity (SD2), it shows beat-to-beat consistency between the initial (*n*) and subsequent (*n* + 1) RR interval. Hence, deviation of the clustered SD2 points away from the average RR interval, taken with reference to the centroid, represents long-term variability. The ratio SD2 to SD1 then gives an indication of the degree of long-term variability in relation to the short-term variability.

Coined in 1991 by Pincus et al., the concept of approximate entropy was introduced to provide approximations on the degree of regularity when applied to a short-duration epoch, which cannot be achieved with moment statistics such as mean and variance. This is applied to non-stationary biomedical data such as heart rate variability, which commonly presents with non-linearity and complexity. Logarithmically, the approximate entropy takes into account the imputed threshold “*r”* under which a recurrence is identified. With this it expresses the likelihood of repeated signals within the threshold for *m* and *m* + 1 points. It is computed as follows:

Firstly, a set of length m vectors u_j_ is formed:

(7) $${\text{u}}_{\text{j}}=({\text{APD}}_{\text{j}};{\text{APD}}_{\text{j+1}},\ldots ,{\text{APD}}_{\text{j+m}-\text{1}});\text{j=1};\;2;\ldots \text{N}-\text{m}+\text{1}$$

where, m is the embedding dimension and N is the number of measured APDs. The distance between these vectors is defined as the maximum absolute difference between the corresponding elements:

(8) $$\text{d}({\text{u}}_{\text{j}},{\text{u}}_{\text{k}})=\text{max}\{{\text{|APD}}_{\text{j+n}}-{\text{APD}}_{\text{k+n}}\text{||n}=\text{0},\ldots ,\text{m}-\text{1}\}$$

for each u_j_ the relative number of vectors u_k_ for which d(u_j_, u_k_) ≤ r is calculated. This index is denoted with Cmj (r) and can be written in the form

(9) $$C_{j}^{m}\left(r\right)=\;\frac{\text{nbr of}\left\{u_{k}|\;d\left(u_{j},u_{k}\right)\leq r\right\}}{N-m+1}\;\forall k$$

Taking the natural logarithms gives:

(10) $$\Phi^{m}\left(r\right)=\frac{1}{N-m+1}\sum_{j=1}^{N-m+1}\text{ln}C_{j}^{m}\left(r\right).$$

The approximate entropy is then defined as:

(11) $$\text{ApEn}\left(m,\;r,\;N\right)=\Phi^{m}\left(r\right)-\Phi^{m+1}(r)$$

Approximate entropy measures the likelihood that certain patterns of observations are followed by different patterns of observations. As such, a lower approximate entropy values reflect a more regular signal, whereas higher values reflect a more irregular signal (Pincus, 1991; Mesin, 2018).

The sample entropy also provides a measure of signal irregularity but is less susceptible to bias than approximate entropy (Richman and Moorman, 2000; Nayak et al., 2018). This is done by eliminating the counting of self-matches; hence the count of the number of similar vector lengths is always one less than that of ApEn. Furthermore, sample entropy uses the logarithm of the sum of conditional properties rather than each conditional property individually, illustrated by the negative natural logarithm for conditional properties. Both sample entropy and approximate entropy are able to differentiate between experimental and theoretical data sets. However, it has been demonstrated that sample entropy yielded better relative consistency compared to approximate entropy, reflecting independence from data length and choice of *m* or *r* (Molina-Pico et al., 2011).

This is given by:

(12) $$C_{j}^{m}\left(r\right)=\frac{\text{nbr of}\left\{u_{k}|\;d\left(u_{j},u_{k}\right)\leq r\right\}}{N-m}\;\forall k\;\neq j$$

Averaging then gives:

(13) $$C^{m}\left(r\right)=\;\frac{1}{N-m+1}\;\sum_{j=1}^{N-m+1}C_{j}^{m}(r)$$

The sample entropy is then given by:

(14) $$\text{SampEn}\left(m,\;r,\;N\right)=\;\ln(\frac{C^{m}\left(r\right)}{C^{m+1}\left(r\right)})$$

Finally, detrended fluctuation analysis (DFA) was performed to determine long-range correlations in non-stationary physiological time series (Peng et al., 1995), yielding both short-term fluctuation (α1) and long-term fluctuation (α2) slopes. The point at which the slopes α1 and α2 is the crossover point.

### Statistical analysis

All values were expressed as mean ± standard error of the mean (SEM). Numerical data were compared by one-way analysis of variance (ANOVA), a statistical technique that utilizes the F-distribution to compare the means or two or more samples. *P* < 0.05 was considered statistically significant and was denoted by ^*^ in the figures.

## Results

### Atrial and ventricular action potential duration variability determined using time-domain and frequency-domain methods

**Figure 1:**
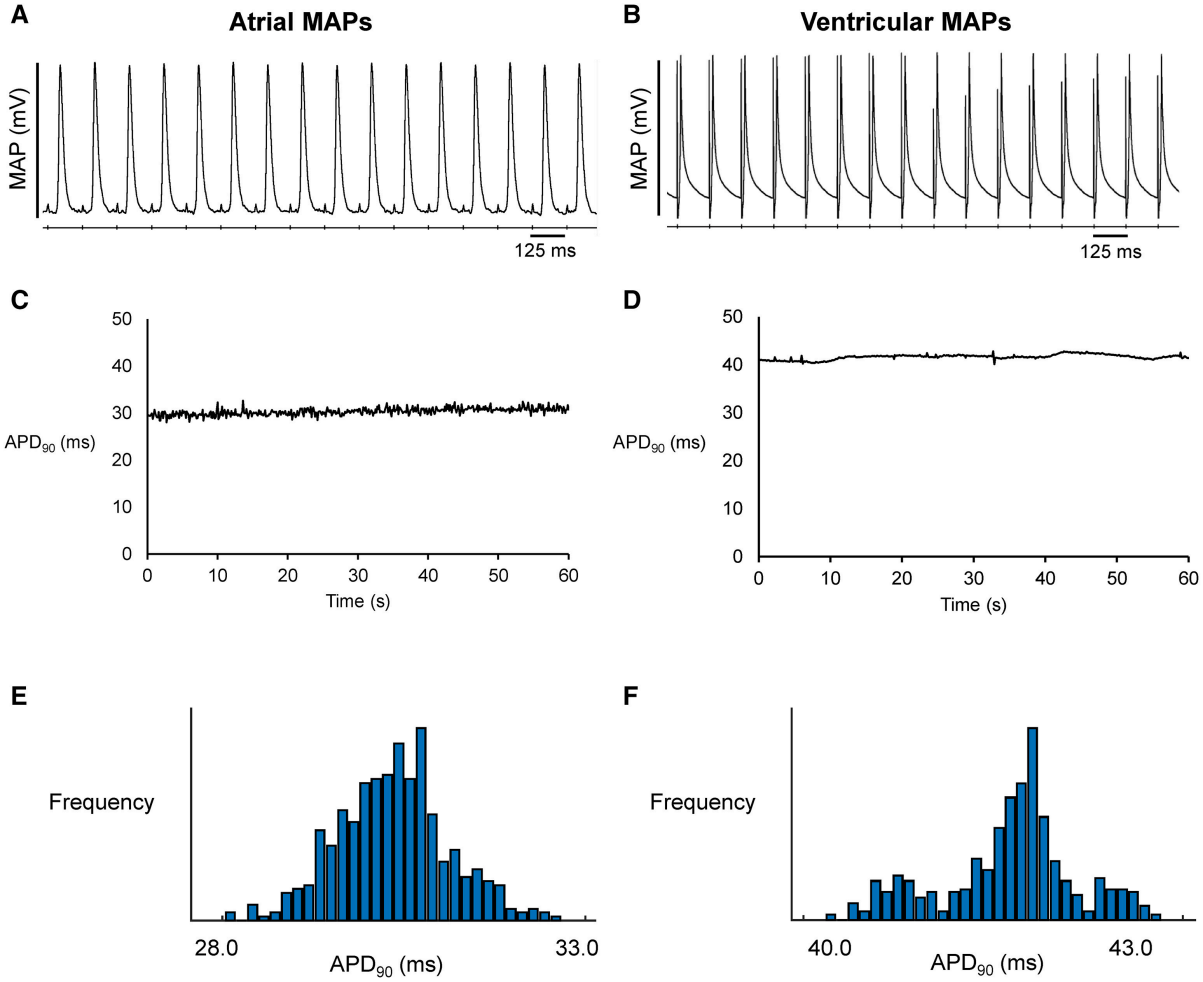
Representative MAP traces from a single heart obtained over a ten-second period during regular 8 Hz pacing from the left atrium **(A)** or left ventricle **(B)**. The corresponding time-series **(C,D)** and histograms **(E,F)** for action potential duration at 90% repolarization (APD_90_).

**Figure 2:**
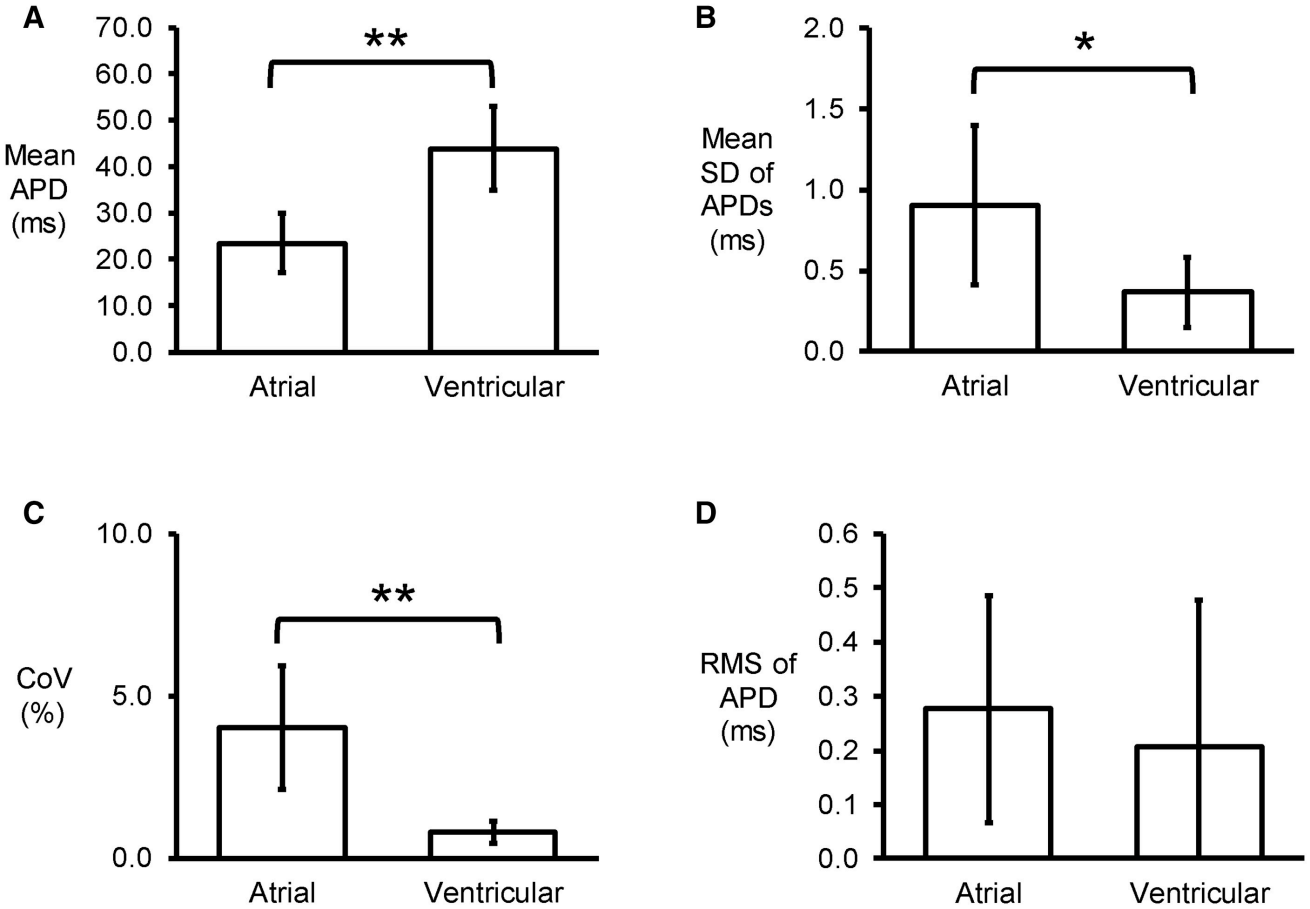
Time-domain analysis yielding mean APD **(A)**, standard deviation (SD) of APDs **(B)**, coefficient of variation (CoV) **(C)**, and root mean square (RMS) of successive differences of APDs **(D)** (*n* = 6; ^*^P < 0.05; ^**^P < 0.01).

Representative stable MAP recordings were obtained from the left atrial (Figure 1A) or ventricular (Figure 1B) epicardium of Langendorff-perfused mouse hearts during regular 8 Hz pacing. Typical time series of atrial and ventricular APDs at 90% repolarization (APD_90_) are shown in Figures 1C,D, respectively and their corresponding histograms are shown in Figures 1E,F, respectively. Atrial APD_90_ took a mean value of 23.5 ± 6.3 ms (Figure 2A) with a mean standard deviation (SD) 0.9 ± 0.5 ms (Figure 2B) (*n* = 6 hearts). The coefficient of variation (CoV), a measure of relative variability calculated by dividing SD by the mean and subsequently multiplying by 100%, was 4.0 ± 1.9% (Figure 2C) and the root mean square (RMS) of successive differences in APDs was 0.3 ± 0.2 ms (Figure 2D). By contrast, ventricular APD_90_ (*n* = 6 hearts) were longer than atrial APD_90_ (44.0 ± 9.1 ms; ANOVA, *P* < 0.05), with lower mean SD (0.4 ± 0.2 ms, *P* < 0.05), CoV (0.8 ± 0.3%, *P* < 0.01) but similar RMS of successive differences in APD_90_ (0.2 ± 0.3%, *P* > 0.05).

**Figure 3:**
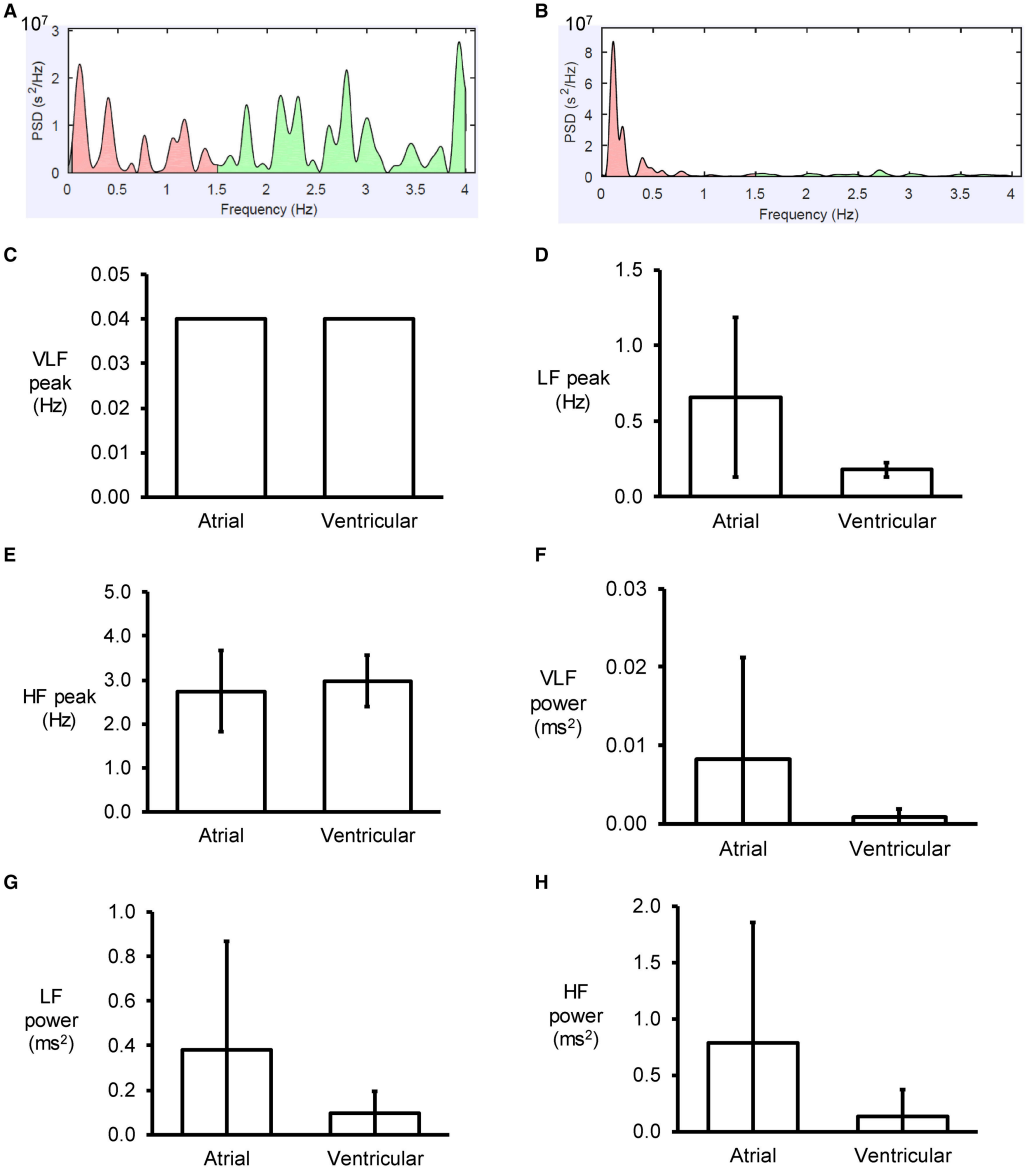
Examples of frequency spectra using the Fast Fourier Transform method for atrial **(A)** and ventricular **(B)** MAP recordings. Peaks for very low- **(C)**, low- **(D)** and high-frequency **(E)** for atrial and ventricular MAPs, and their percentage powers **(F–H)**.

An example of a frequency spectrum using the Fast Fourier Transform method is shown in Figure 3A. Frequency-domain analysis revealed that the peaks for very low-, low- and high-frequency for atrial MAPs were 0.04 ± 0.00, 0.7 ± 0.5 and 2.7 ± 0.9 Hz, respectively (Figures 3B–D), with percentage powers of 1.7 ± 2.6, 39.0 ± 20.5, and 59.3 ± 22.9% (Figures 3E–G). For the ventricles, similar peak frequencies (0.04 ± 0.00, 0.2 ± 0.0 and 3.0 ± 0.6%) and percentage powers (0.9 ± 1.1, 66.0 ± 27.8, and 32.5 ± 27.0) were observed (ANOVA, *P* > 0.05).

**Figure 4:**
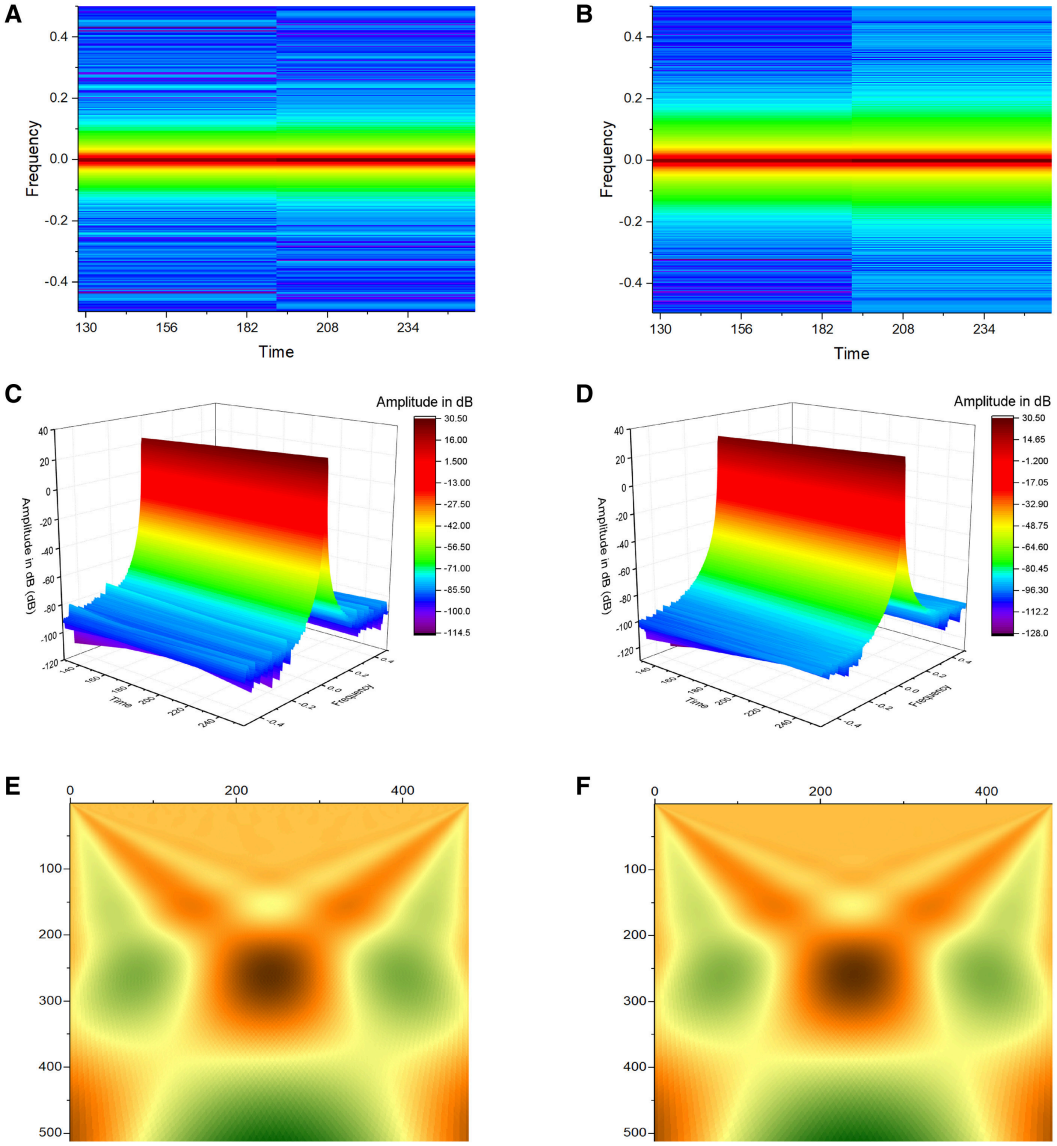
Application of Short-Time Fourier Transform (STFT) yielded plots demonstrating frequency against time for atrial **(A)** and ventricular APD_90_ **(B)**, and their corresponding three-dimensional representations **(C,D)**. Continuous wavelet transform (CWT) with Morlet wavelets as basis functions of atrial **(E)** and ventricular APD_90_ **(F)**.

Simultaneous time-frequency analysis was subsequently performed using short-time Fourier transform (STFT) and continuous wavelet transform (CWT). Application of STFT yielded plots demonstrating frequency against time for atrial and ventricular APD_90_ (Figures 4A,B), and their corresponding three-dimensional representations (Figures 4C,D). CWT with Morlet wavelets as basis functions of atrial and ventricular APD_90_ yielded image plots shown in Figures 4E,F, respectively.

### Action potential duration variability determined using non-linear methods

**Figure 5:**
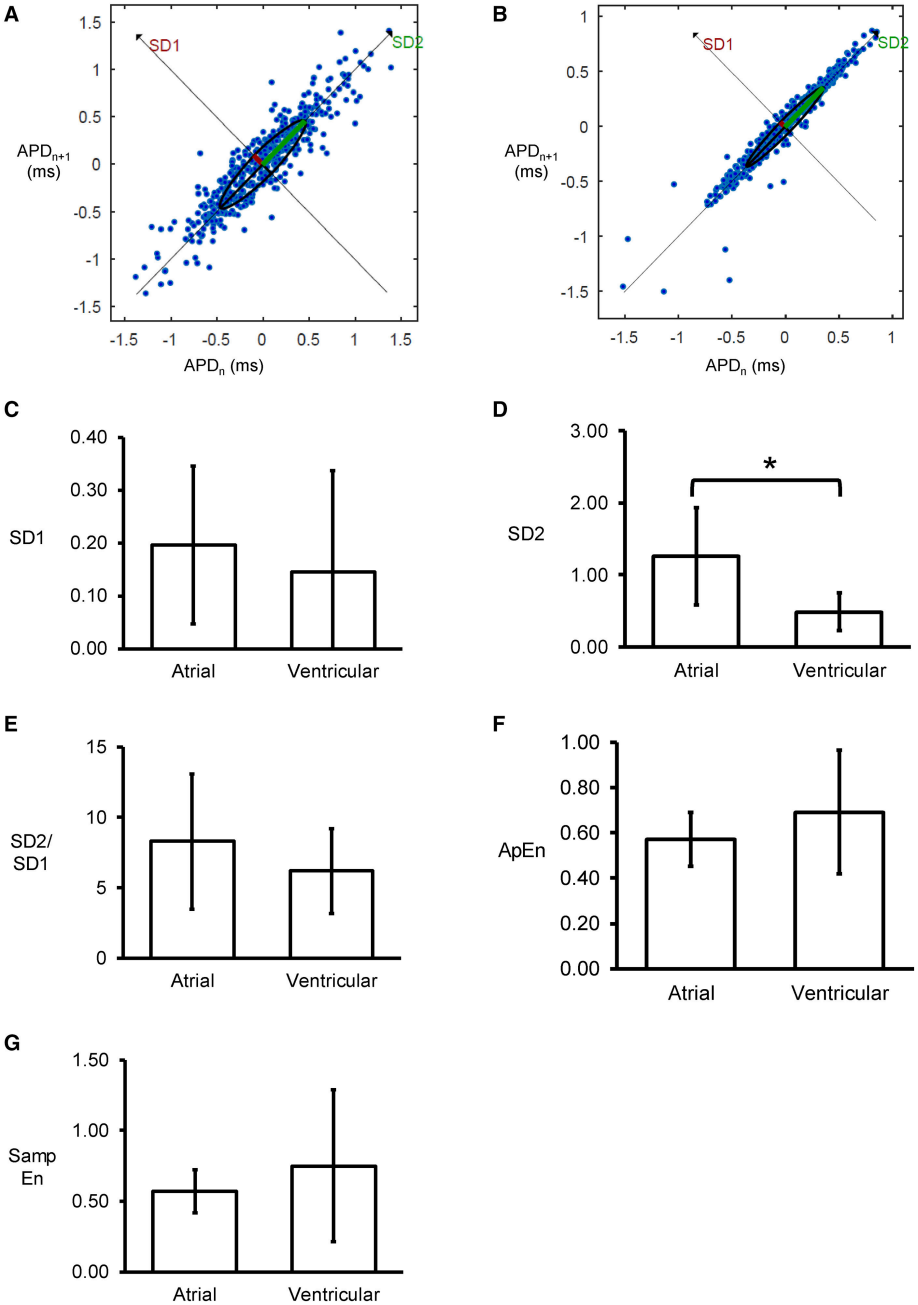
Representative Poincaré plots of APD_n+1_ against APD_n_ from the left atrium **(A)** or left ventricle **(B)** from a single heart. SD along the line-of-identity (SD1) **(C)** and SD perpendicular to the line-of-identity (SD2) **(D)**, and the SD2/SD1 ratio **(E)**, approximate entropy **(F)**, and sample entropy **(G)** (^*^P < 0.05).

Poincaré plots expressing APD_n+1_ as a function of APD_n_ were constructed for the atrial and ventricular MAPs (Figures 5A,B). In all of the hearts studied, ellipsoid shapes of the data points were evident. The SD perpendicular to the line-of-identity (SD1) and SD along the line-of-identity (SD2) are shown in Figures 5C,D, respectively. For atrial recordings, the mean SD1 and SD2 were 0.20 ± 0.15 and 1.26 ± 0.67, respectively. The SD2 to SD1 ratio took a mean value of 8.28 ± 4.78 (Figure 5E). The approximate and sample entropy took values of 0.57 ± 0.12 (Figure 5F) and 0.57 ± 0.15 (Figure 5G), respectively. For ventricular MAPs, Poincaré plots of APD_n+1_ against APD_n_ revealed similar ellipsoid shapes. They showed similar SD1 (0.15 ± 0.19, *P* > 0.05) and lower SD2 (0.49 ± 0.26, *P* < 0.05). Nevertheless, there was no difference in SD2/SD1 ratio (6.19 ± 3.03, *P* > 0.05). Moreover, approximate entropy (0.69 ± 0.27, *P* > 0.05), and sample entropy (0.75 ± 0.54, *P* > 0.05) were statistically indistinguishable when compared to the atrial parameters.

**Figure 6:**
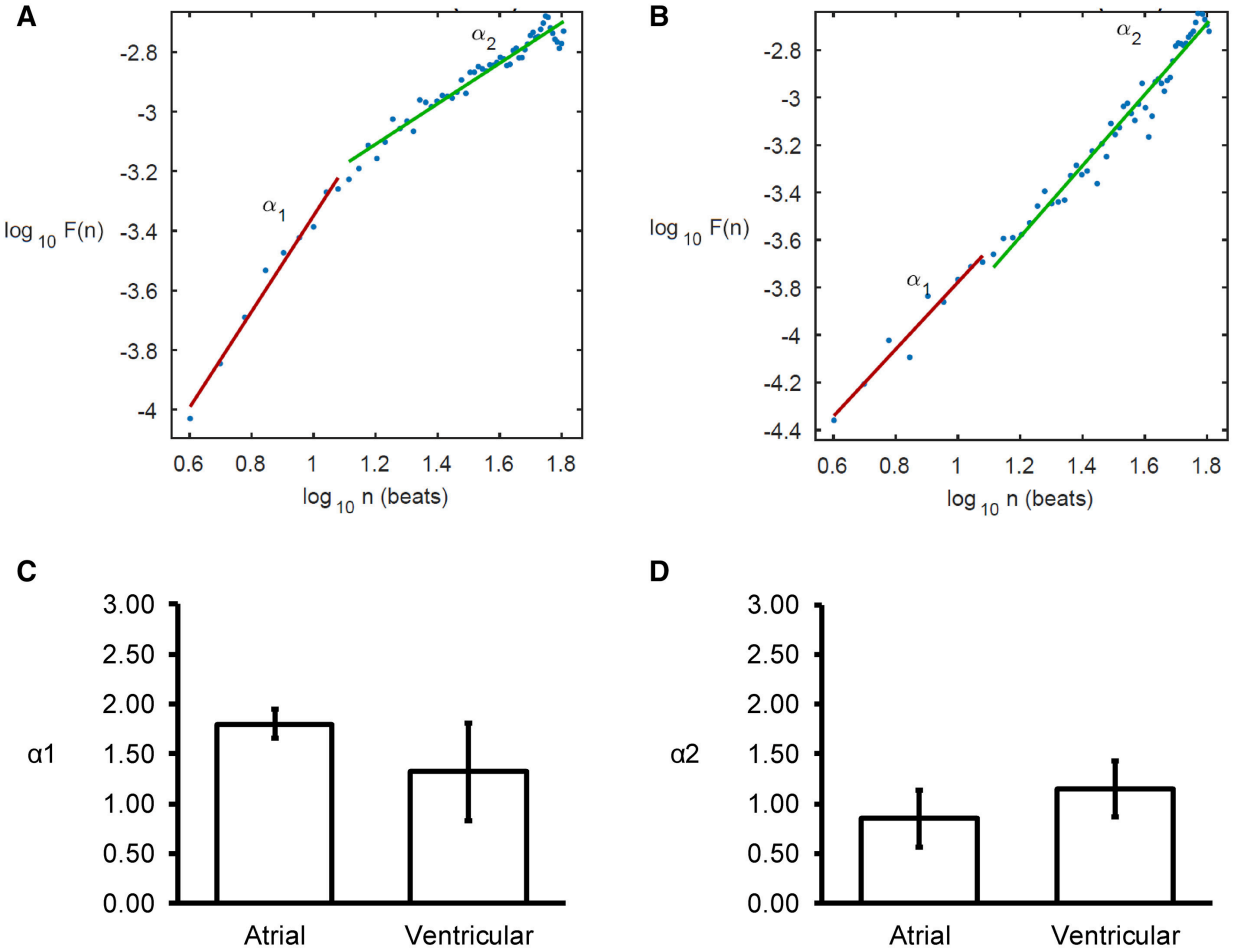
Detrended fluctuation analysis (DFA) plots expressing detrended fluctuations F(n) as a function of n in a log-log scale for the atria **(A)** and ventricles **(B)**, yielding short-term **(C)**, and long-term **(D)** fluctuation slopes (α1 and α2, respectively).

Detrended fluctuation analysis plotting the detrended fluctuations F(n) as a function of n in a log-log scale was performed for the atrial and ventricular MAPs (Figures 6A,B). This revealed short- (α1) and long-term (α2) fluctuation slopes of 1.80 ± 0.15 (Figure 6C) and 0.85 ± 0.29 (Figure 6D), respectively for the atria, which were not significantly different from the values obtained from the ventricles (1.32 ± 0.49 and 1.15 ± 0.28, respectively, both *P* > 0.05). α1 was significantly larger than α2 in the atria (ANOVA, *P* < 0.001) but not in the ventricles (ANOVA, *P* > 0.05).

The variability data for APD_70_, APD_50_, and APD_30_ are shown in Supplementary Appendices 1–3, respectively.

## Discussion

This is the first proof-of-concept study investigating the beat-to-beat variability in repolarization time-courses of atrial and ventricular MAP recordings in whole hearts of mice. The main findings are that (1) variability in APDs can be detected using time-domain, frequency-domain, combined time-frequency, and non-linear methods; (2) the atria and ventricles show similar low- and high-frequency peaks; (3) but the atria showed predominantly low-frequency components whereas the ventricles showed predominantly high-frequency components; (4) Poincaré plot showed ellipsoid shapes from all of the hearts; (5) the SD perpendicular to the line-of-identity (SD2) was significantly larger than the SD along the line-of-identity (SD1), leading to SD2/SD1 ratios greater than unity; (6) a degree of disorder was identified by approximate and sample entropy analyses, (7) short-term fluctuation slopes were steeper than long-term fluctuation slopes.

Variability in recorded signals is an intrinsic property of excitable media in biological systems. In the heart, heart rate variability (HRV) is normally observed in the healthy state (Shaffer and Ginsberg, 2017), whereas alterations in HRV have been associated with adverse outcomes such as arrhythmogenesis that may be mediated through generation of APD variability (Mcintyre et al., 2014). Similarly, beat-to-beat variability in the repolarization time-course can be present and can be observed electrocardiographically as QT interval variability (Baumert et al., 2016; Orini et al., 2016). Naturally occurring beat-to-beat variations in APDs have been observed in isolated cardiomyocytes (Kiyosue and Arita, 1989; Shryock et al., 2013), even when pacing rate and temperature are held constant (Zaniboni et al., 2000). It has been studied in detail in canine ventricular cardiomyocytes (Abi-Gerges et al., 2010; Kistamas et al., 2015a,b; Szentandrassy et al., 2015; Magyar et al., 2016), but never in mouse models whether in single cells or isolated hearts. Our study adds to the literature by demonstrating that such variabilities are also present in Langendorff-perfused mouse hearts under similar constant rate pacing conditions. Computational modeling has previously identified the molecular mechanisms underlying such beat-to-beat variability in APDs (Heijman et al., 2013). These include stochastic gating of ion channels, in particular that of sodium and delayed rectifier potassium channels. Although fluctuations in APDs was present in our experimental mouse model, the variability was very small, with standard deviation of around 1.4 ms for the atria and 0.2 ms for the ventricles. This may be due to the differing morphology of the cardiac action potentials in this species. Consistent with these findings, modeling studies suggests that variability is higher in species that have more pronounced plateau phase during repolarization, such as guinea pigs and rabbits (Heijman et al., 2013), than those with a triangular action potential morphology such as mice. Indeed, the standard deviation is around 10 ms in guinea pig ventricular cardiomyocytes (Zaniboni et al., 2000) and 7 ms in rabbit sinoatrial nodal cells (Wilders and Jongsma, 1993). This variability is dependent on the APD. Therefore, one way to express this is the coefficient of variation (CoV), given by the percentage of SD divided by the mean APD. The CoV is around 2% in both the guinea pig and the rabbit ventricles. From our study, we found CoV to be 4.0% in the atria and 0.8% in the ventricles. It should be noted that our model used intact hearts whereas single cells were used in the other studies. Multicellular preparations are known to show lower levels of variability than in single cells because of electrical coupling, which dampens the differences between cells (Magyar et al., 2015).

Time-domain analysis allowed the quantification of the variability using standard deviations, coefficients of variations and root mean squares of successive APDs in both the atria and ventricles. It was noted that atrial APDs were significantly shorter than ventricular APDs, in keeping with our previous findings (Tse et al., 2016a,b). Moreover, we report for the first time higher degrees of variability in the atria as reflected by higher mean SD, CoV and RMS of APDs when compared to the ventricles. Frequency-domain analysis using the Fast Fourier Transform-based method produced power spectrum density estimates for the APD_90_ time series. This provides the basic information on how power is distributed as a function of frequency. We observed that both atrial and ventricular MAPs were predominantly in the low-frequency domain. LF and HF rhythms in repolarization variability are important as they reflect QT rate adaptation (Merri et al., 1993). Variability assessed in the frequency domain represents an index of temporal dispersion of ventricular repolarization (Lombardi et al., 1998) which is an important determinant of arrhythmogenesis. However, the above frequency analysis does not provide any information on the time evolution of the frequencies. To achieve, this, time-frequency analysis was conducted using both short-time Fourier transform (STFT) and continuous wavelet transform (CWT). Previously, time-frequency analysis has been applied to electrograms to detect regional cardiac repolarization alternans that occur transiently (Orini et al., 2013, 2014).

Significantly, non-linear analyses of APDs yielded further insights. Thus, Poincaré plots of APDs showed ellipsoid shapes in all of the hearts studied, and together with a SD2/SD1 ratio >1, indicated that variability in the long-term was greater than variability in the short-term. This ratio was around 6 to 8 and did not significantly differ between the atria and ventricles. In a canine model, higher short-term variability calculated from Poincaré plots being associated with the occurrence of drug-induced *torsade de pointes* (Thomsen et al., 2004). Furthermore, the present findings also found a degree of entropy present in the atria and ventricles. Entropy refers to the degree of disorder in a system and has been used to quantify the regularity or complexity of biological signals (Pincus, 1991; Pincus and Goldberger, 1994). These entropy calculations are based on the state space reconstruction of time series data (Richman and Moorman, 2000; Bandt and Pompe, 2002; Li et al., 2015). Our study quantified for the first time approximate entropy in the atria and ventricles. This is an appropriate method for time series with more than 50 points, a condition that we have satisfied (Pincus, 2001). Similar, this study determined sample entropy, which is a refined version of approximate entropy. It can quantify the irregularity of APD time series without biasing (Richman and Moorman, 2000) and has the advantage of eliminating self-matches and being less dependent on time-series length (Li et al., 2009). Entropy has been identified as a pro-arrhythmic indicator (Cervigon et al., 2016). High entropy in repolarization was shown to predict arrhythmic or mortality outcomes in patients receiving implantable-cardioverter defibrillator for primary prevention of sudden cardiac death (Demazumder et al., 2016). Further studies are needed to confirm or refute the hypothesis that increased approximate or sample entropy predicts the onset of atrial or ventricular arrhythmias in mouse hearts. However, its use has some important limitations. For example, it should not be applied to long duration signals because more computations are required for real-time implementation (Tripathy et al., 2017).

Fractional calculus has been applied to investigate physiological time series such as heart rate variability (González et al., 2012; Sturmberg and West, 2013; Sturmberg et al., 2015). Some techniques assume stationary signals whilst others do not make such assumptions (Gao et al., 2013). This study applied for the first time detrended fluctuation analysis (DFA) to reveal complex fractal fluctuation patterns by delineating them into long- and short-term fluctuation for the first time in the mouse heart. DFA is a method for quantifying long-range correlations in non-stationary physiological time series (Peng et al., 1995). DFA enables correct estimation of the power law scaling, the Hurst exponent, in the presence of extrinsic non-stationaries while eliminating spurious detection of long-range dependence (West et al., 2008). The average fluctuation is plotted against the number of beats on a log-log scale, yielding short- and long-term fluctuation slopes, or scaling exponents (α1 and α2, respectively). α of 0.5 indicates uncorrelated data, and deviations from 0.5 indicates the presence of correlation. For example, in the atria, we found α1 to be around 1.7, suggesting the presence of short-term correlation, but α2 was around 0.7, suggesting the minimal long-term correlations. In the ventricles, α1 and α2 took similar values to those observed in the atria.

Previously, decreases in the short-term exponent of HRV, has been associated with arrhythmic and mortality outcomes in heart failure after acute myocardial infarction (Huikuri et al., 2000) and in end-stage renal failure patients receiving peritoneal dialysis (Chiang et al., 2016). Decreases in the short-term exponent have also been detected prior to the onset of atrial arrhythmias (Vikman et al., 1999). In a rabbit hypertrophic cardiomyopathy model, DFA of maximum QT intervals showed higher scaling exponent in diseased compared to control groups (Sanbe et al., 2005). In human induced pluripotent stem cell-derived cardiomyocytes, fractal correlations as determined by α1 was observed (Kuusela et al., 2016). In humans, a significant decrease in α1 was observed during sympathetic activation suggesting a breakdown of the short-term fractal organization of heart rate (Tulppo et al., 2005). Moreover, normal α1 but lower α2 was observed in patients with atrial fibrillation compared to those without AF (Kalisnik et al., 2015).

Previous work has demonstrated that HRV time series have a crossover phenomenon (Havlin et al., 1999; Penzel et al., 2003). In this study, DFA also found scaling trends with two distinct values. This is interesting because it may be related to bi-fractality, where fractal patterns can emerge from random fluctuations via allometric filtering mechanisms (Scafetta and West, 2007). Thus, APD time series are potentially crossover-fractals with two fractal dimensions. This could be validated by using empirical mode decomposition to construct crossover-fractals from two monofractals (Liaw and Chiu, 2010). However, although DFA is useful for exploring the structure of correlations in physiological time series, tracking the local evolution of the exponent by a recursive least-squares method can yield structures of correlations that can provide additional details on the dynamics of these series (Bojorges-Valdez et al., 2007). Our findings suggest that repolarization characteristics exhibit fractal behavior and may be better represented using concepts from fractional calculus, for example by using fractal dynamical equations (Marculescu and Bogdan, 2011). Such an approach has successfully been used to optimize control for implantable pacemakers (Bogdan et al., 2012, 2013).

Moreover, fractional differintegration was used to characterize HRV, allowing determination of the standard deviation of the fractionally differintegrated RR time series for a fractional differintegration of order α [SDFDINN(α)]. α_c_, the order of the fractional differintegration that provide the minimum standard deviation of the fractionally differintegrated RR set, showed a linear correlation with the Hurst exponent. Interestingly this method for estimating the exponent showed less bias and lower variance when compared to DFA (García-González et al., 2013). Also, α_c_ was closely related to α_1_ but they were not equal. Future studies are needed to explore the predictive values of these fluctuation exponents, and to evaluate the efficacy of fractal dynamical state equation to describe the spatial and temporal dependency structure of repolarization properties in mouse models of cardiac arrhythmias (Xue and Bogdan, 2017).

## Conclusions

The present findings provide a proof-of-concept that APD variability is present at baseline conditions and can be detected using time-domain, frequency-domain and non-linear techniques. Atrial MAPs showed greater degree of variability than ventricular MAPs. Non-linear techniques offer further insights on short-term and long-term variability and signal complexity.

## Author contributions

GT: study conception, data analysis and interpretation, manuscript drafting, critical revision of manuscript. YD, FC, TL, GH, KHCL, GL, GB, and SC: data interpretation, critical revision of manuscript. KL, WKW, and WTW: study supervision, data interpretation, critical revision of manuscript.

### Conflict of interest statement

The authors declare that the research was conducted in the absence of any commercial or financial relationships that could be construed as a potential conflict of interest.
