# Supplementary material for: Quantification of Beat-To-Beat Variability of Action Potential Durations in Langendorff-Perfused Mouse Hearts
Source: Front Physiol. 2018 Nov 27;9:1578. doi: 10.3389/fphys.2018.01578 (PMC6277547; doi:10.3389/fphys.2018.01578)
Supplement: Supplementary file 1 [file Data_Sheet_1.PDF]

Supplementary Material

Specific MHC-I Peptides are Induced using PROTACs

Authors:

Stephanie M. Jensen, Gregory K. Potts, Damien B. Ready, and Melanie J. Patterson*

Correspondence:

Melanie.Patterson@abbvie.com

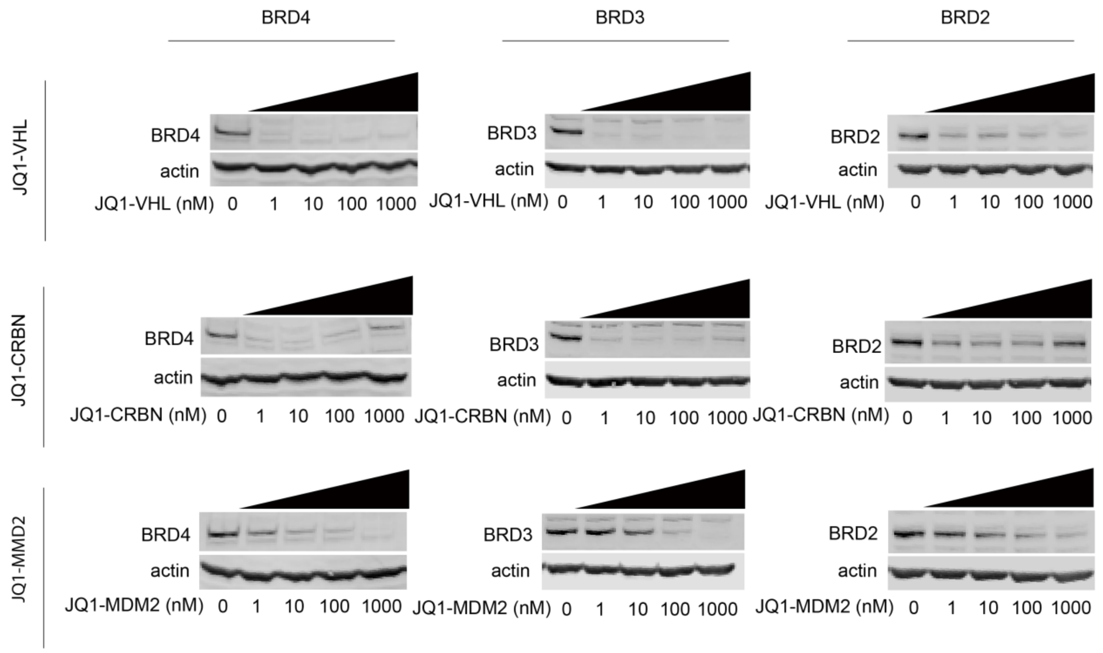

Figure S1. PROTAC compounds induce the degradation of BRD4, BRD3, and BRD2. BV173 cells were treated with 0-1000 nM of each PROTAC for 16 hours. Blots were performed against BRD4, BRD3, and BRD2. JQ1-MDM2, JQ1-CRBN, and JQ1-VHL successfully degraded all three BRD proteins. JQ1-VHL and JQ1-MDM2 show modest selectivity between BET family members. JQ1-CRBN does not induce strong degradation at higher concentrations of PROTAC, demonstrating the previously described "hooking effect" characteristic of saturating concentrations of PROTAC (Huang and Dixit, 2016).

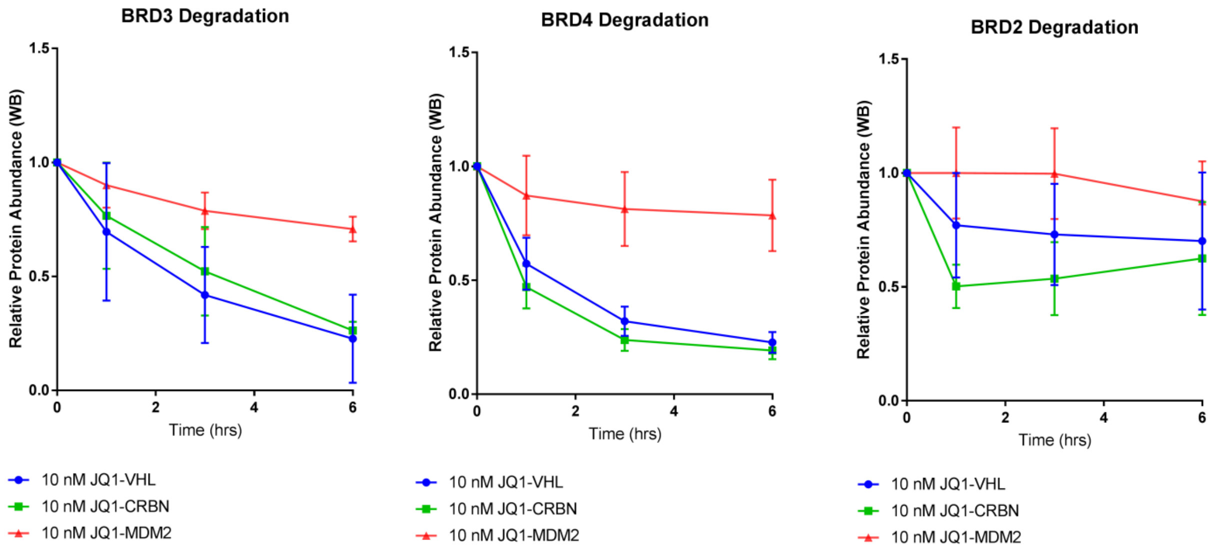

Figure S2. PROTAC compounds show differential degradation of BRD4, BRD3, and BRD2 over a 6 hour time course at 10 nM. BV173 cells were treated with 10 nM of each PROTAC for 0-6 hours. Western blots were performed against BRD4, BRD3, and BRD2. Within a 6 hour timecourse at 10 nM, JQ1-VHL and JQ1-CRBN were the most successful at degrading target proteins, however, both PROTAC compounds only limitedly degraded BRD2. JQ1-MDM2 did not notably degrade BRD2 within 6 hours at 10 nM.

Figure S

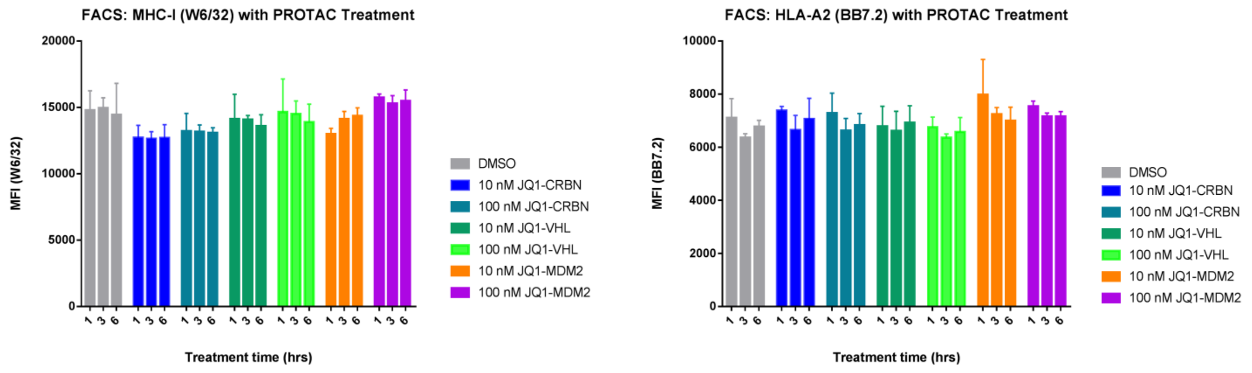

Figure S3. Amount of MHC-I on the cell surface does not change after PROTAC treatment. MHC-I concentration after PROTAC treatment for 0-6 hours as measured by FACS, staining with BB7.2 (HLA-A2) and W6/32 (HLA-A,B,C). Median fluorescence intensity (MFI) was plotted as the average of three replicates. Error bars indicate SD.

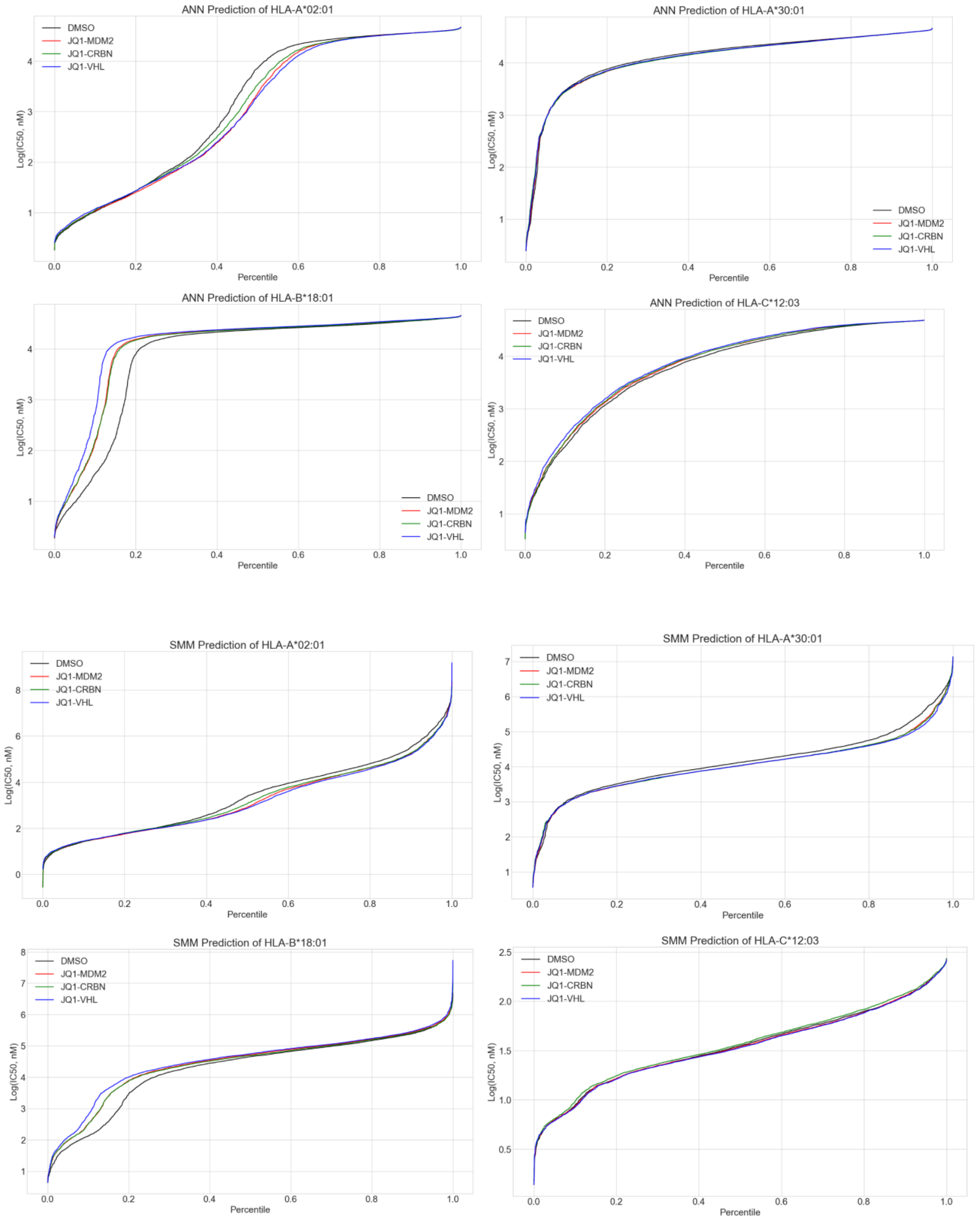

Figure S4. Affinity of all identified MHC-I peptides, analyzed by ANN (top) and SMM (bottom) algorithms. Affinity (SMM and ANN) mapped for single alleles using the IEDB database (only 4 of 6 BV173 alleles were available in IEDB for affinity mapping). Small shifts in mid-range (Log(IC50) = 2-4) affinity were observed in PROTAC-treated samples versus DMSO when analyzed by both SMM and ANN algorithms. These changes were specific to HLA-A2 and HLA-B18 alleles.

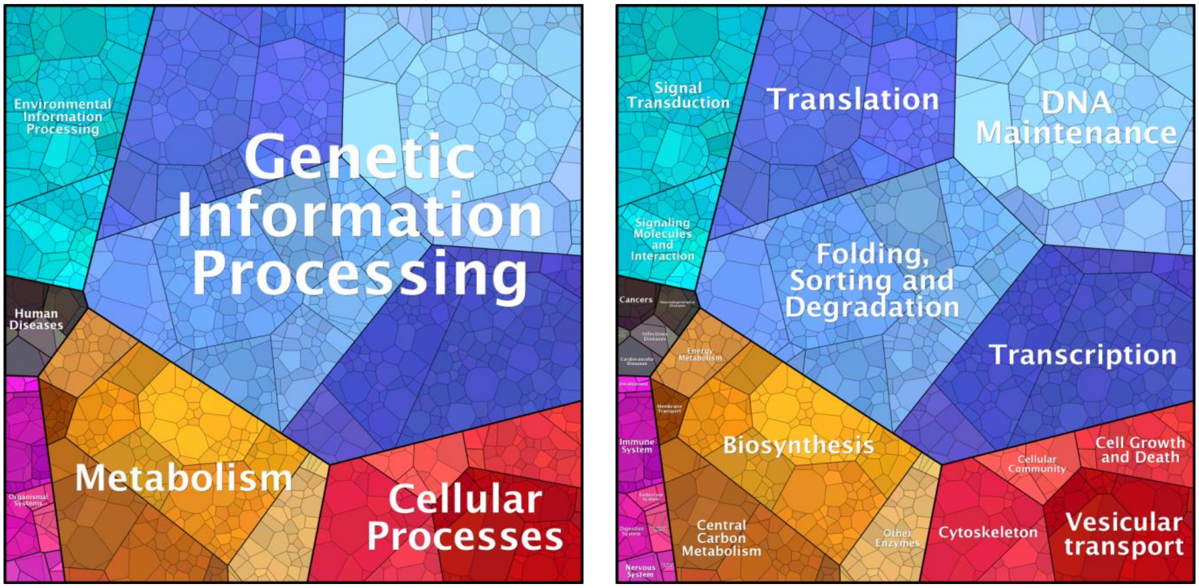

Figure S5: Proteomaps analysis of BV73 MHC-I Immunopeptidome. Proteomaps provides a visual representation of the MHC-I “regular-ome”. The Proteomaps tool assigns KEGG protein annotations to identified proteins. The area of each polygon is equivalent to the number of unique peptide identifications per source protein, multiplied by protein length. This Proteomap was generated from our complete set of MHC-I peptides, isolated across all treatments and time points. This distribution was not found to vary between analyzed samples.

HQVPAVSSV (BRD2)

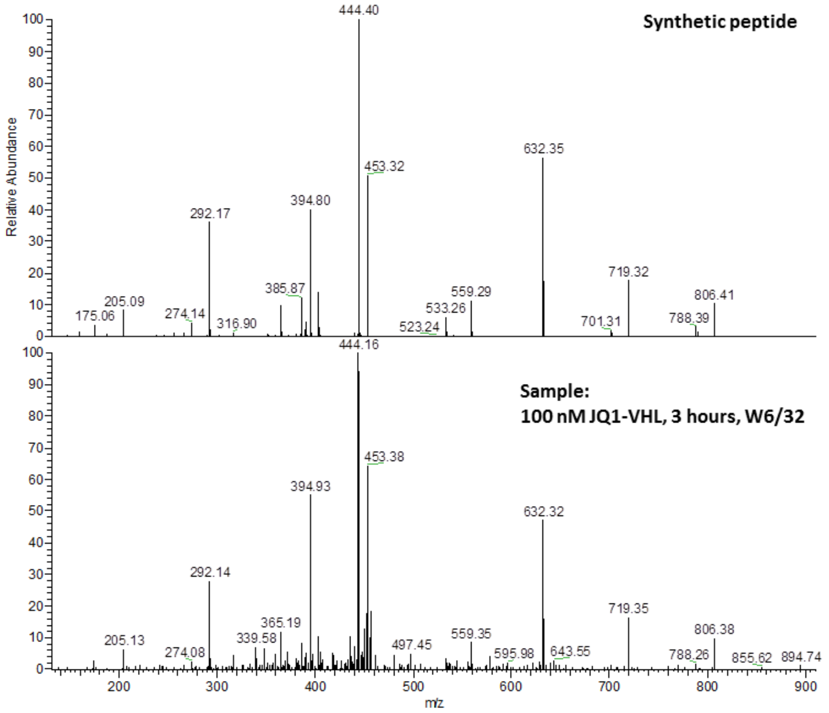

KMPDEPVEA (BRD3)

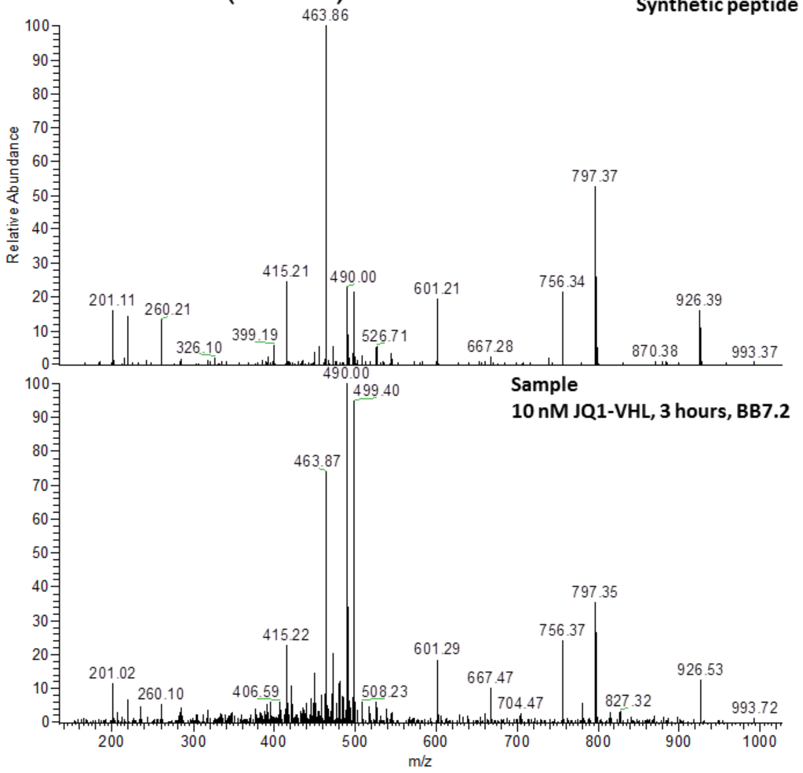

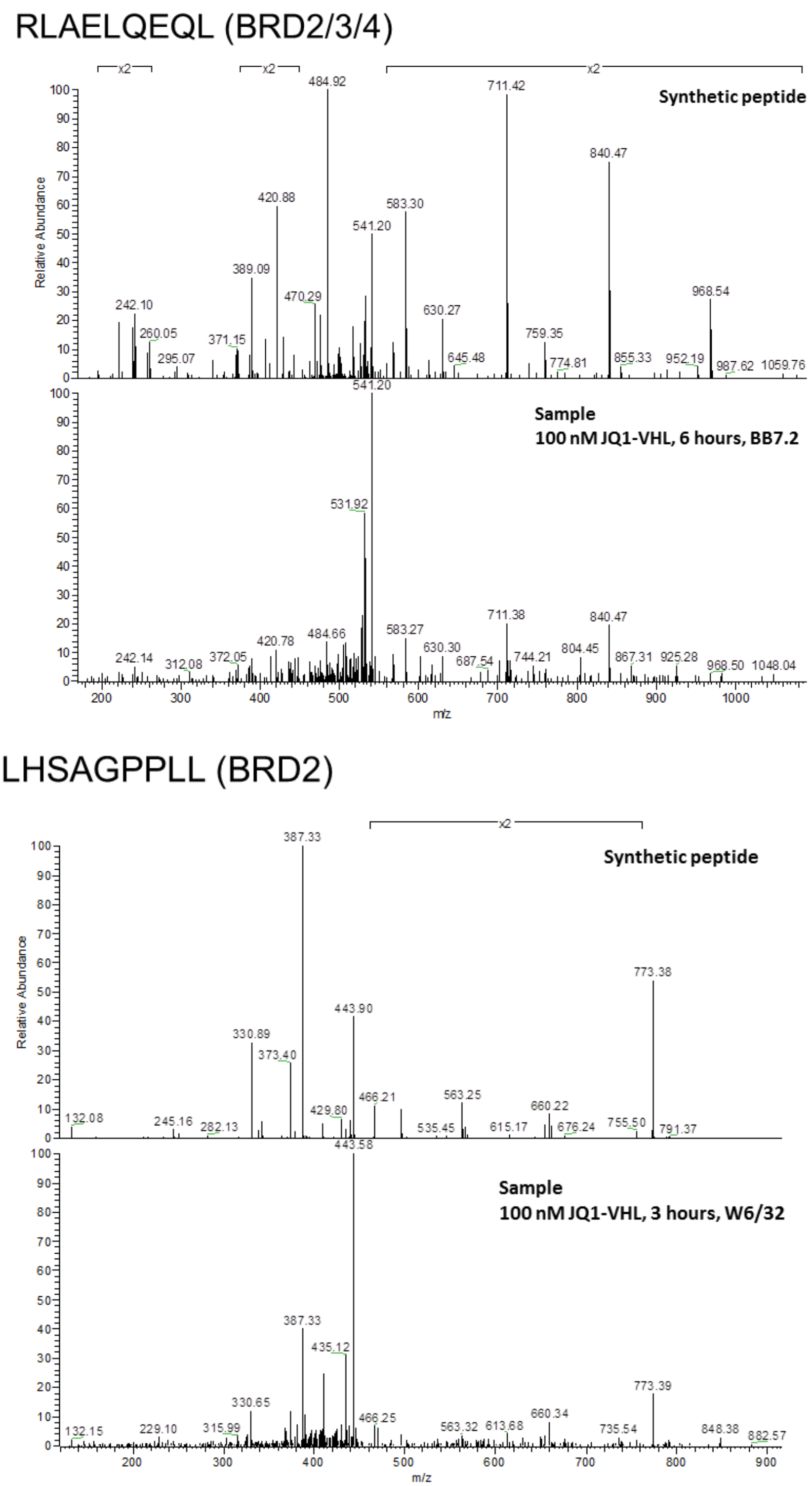

Figure S6. Matching synthetic MS/MS spectra for identified BRD peptides. Synthetic peptides were used to confirm BRD peptide identification using MS/MS CID fragmentation. All identifications were made with <5 ppm precursor mass error and ΔRT <2 minutes.

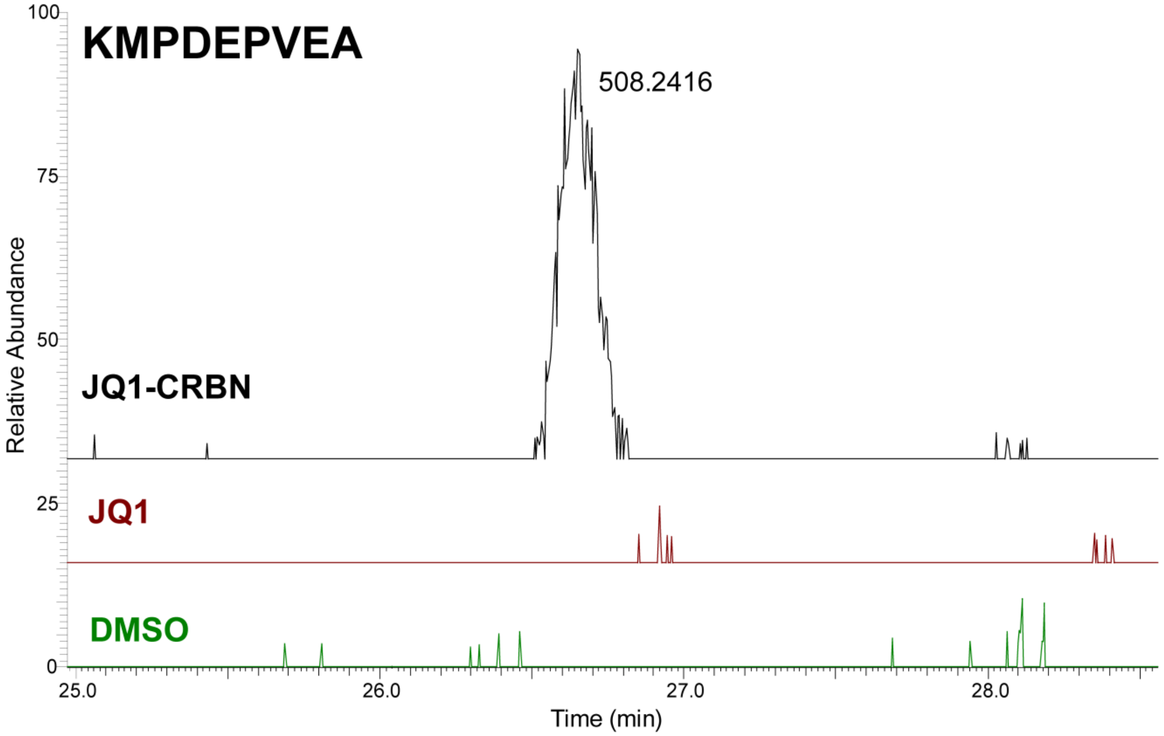

Figure S7. Example XIC comparison indicates BRD peptide presentation is unique to PROTAC treatment. The extracted ion chromatogram (XIC) for KMPDEPVEA peptide (m/z=508.2416, z=2) from a JQ1-CRBN-treated sample, a JQ1-treated sample, and DMSO- (vehicle) treated sample. Peak extraction was performed with a 10 ppm mass tolerance. XIC AUC = $5*10^6$. KMPDEPVEA identification was unique to PROTAC-treated samples.

Sequence	Accession	Gene Name	Description
RLTPKLMEV	O15372	EIF3H	Eukaryotic translation initiation factor 3 subunit H
YLPPATQVV	P38919	EIF4A3	Eukaryotic initiation factor 4A-III
TLLHAGGLARA	P0C7U0	ELFN1	Protein ELFN1
TLFPGKVHSL	Q9NNW5	WDR6	WD repeat-containing protein 6
KILPTLEAV	Q12905	ILF2	Interleukin enhancer-binding factor 2
SLLPEGPPAI	Q15011	HERPUD1	Homocysteine-responsive endoplasmic reticulum-resident ubiquitin-like domain member 1 protein
SLADLQNDEV	P61247	RPS3A	40S ribosomal protein S3a
LLLDVPTAAVQA	P13284	IFI30	Gamma-interferon-inducible lysosomal thiol reductase
NMVAKVDEV	P62906	RPL10A	60S ribosomal protein L10a
VLSSRLAFA	P79483	HLA-DRB3	HLA class II histocompatibility antigen, DR beta 3 chain
TLDEKIEKV	Q96GQ7	DDX27	Probable ATP-dependent RNA helicase DDX27
VMDSKIVQV	O15131	KPNA5	Importin subunit alpha-6
ALSDGVHKI	Q9NVQ4	FAIM	Fas apoptotic inhibitory molecule 1
TLADLVHHV	Q9Y4A5	TRRAP	Transformation/transcription domain-associated protein
LMTKEISSV	P78527	PRKDC	DNA-dependent protein kinase catalytic subunit
TLYEAVREV	P62906	RPL10A	60S ribosomal protein L10a
LLDEPTNHL	Q9UG63	ABCF2	ATP-binding cassette sub-family F member 2
SLNGLEVHL	Q9NQG7	HPS4	Hermansky-Pudlak syndrome 4 protein
QLAQFVHEV	A8MPP1	DDX11L8	Putative ATP-dependent RNA helicase DDX11-like protein 8
ALSDHHIYL	P04075	ALDOA	Fructose-bisphosphate aldolase A
KVAPAPAVV	P62424	RPL7A	60S ribosomal protein L7a
YTSPVNPAV	P61165	TMEM258	Transmembrane protein 258
FLSEEGGHVAV	Q16877	PFKFB4	6-phosphofructo-2-kinase/fructose-2,6-bisphosphatase 4
RLQESVMEA	O60216	RAD21	Double-strand-break repair protein rad21 homolog
TLTEEGVIKV	Q9BZE4	GTPBP4	Nucleolar GTP-binding protein 1
SMSADVPLV	P12004	PCNA	Proliferating cell nuclear antigen
KLDAFVEGV	P21283	ATP6V1C1	V-type proton ATPase subunit C 1
GLIDRQVTV	Q9UPN3	MACF1	Microtubule-actin cross-linking factor 1, isoforms 1/2/3/5
FLDPNNIPKA	Q9BVK2	ALG8	Probable dolichyl pyrophosphate Glc1Man9GlcNAc2 alpha-1,3-glucosyltransferase

Table S6. Housekeeping peptide list: 29 peptides and source proteins used for sample abundance (XIC AUC) normalization.

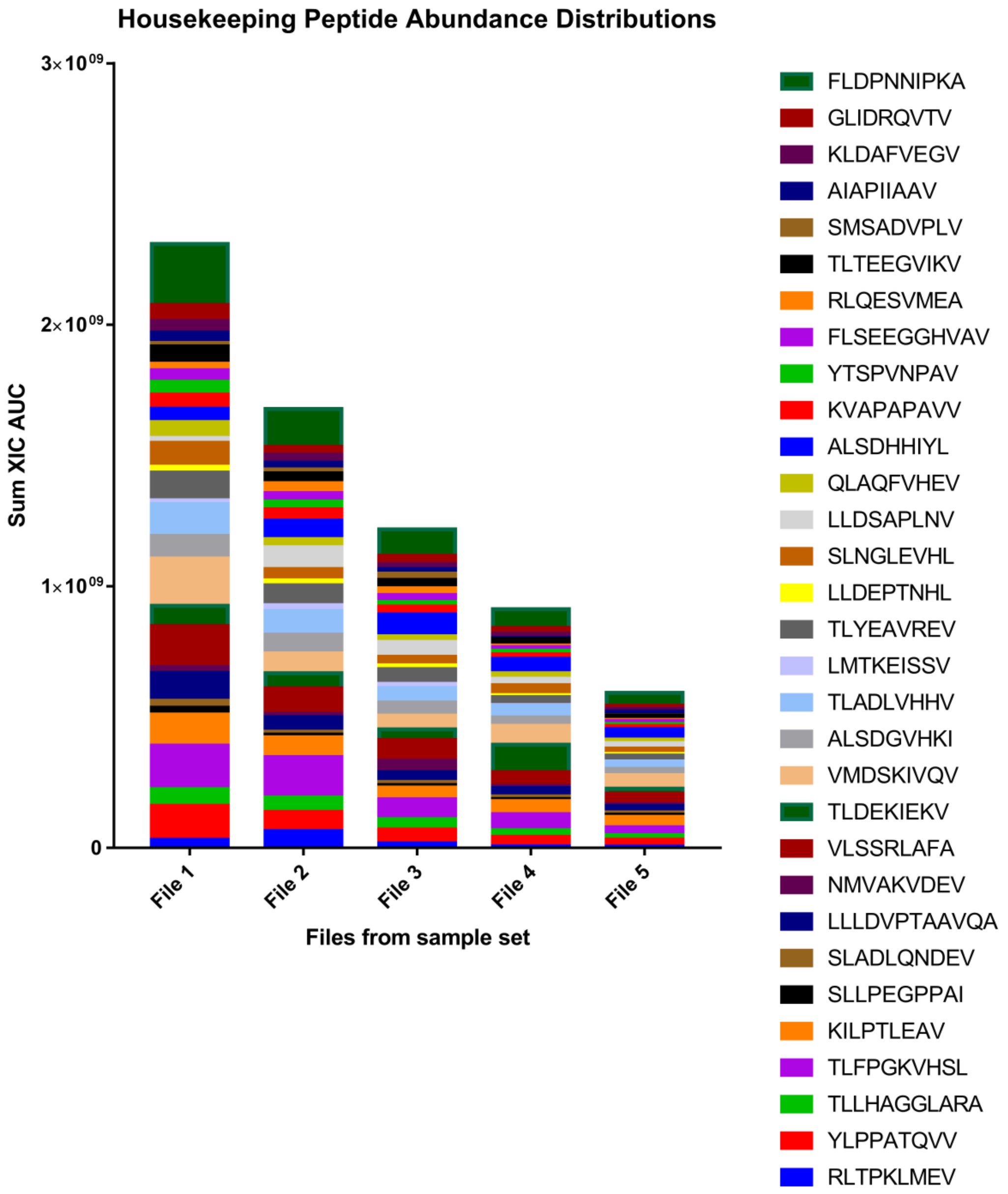

Figure S8. Housekeeping abundance distributions between samples. Samples produced a range of summed abundances across the raw MS data files acquired, depending on sample to sample IP yield and instrument performance. "Housekeeping" peptides used for normalization in our label free quantification experiments showed a proportionate distribution of individual abundances (XIC AUC) with total (sum) sample abundance.

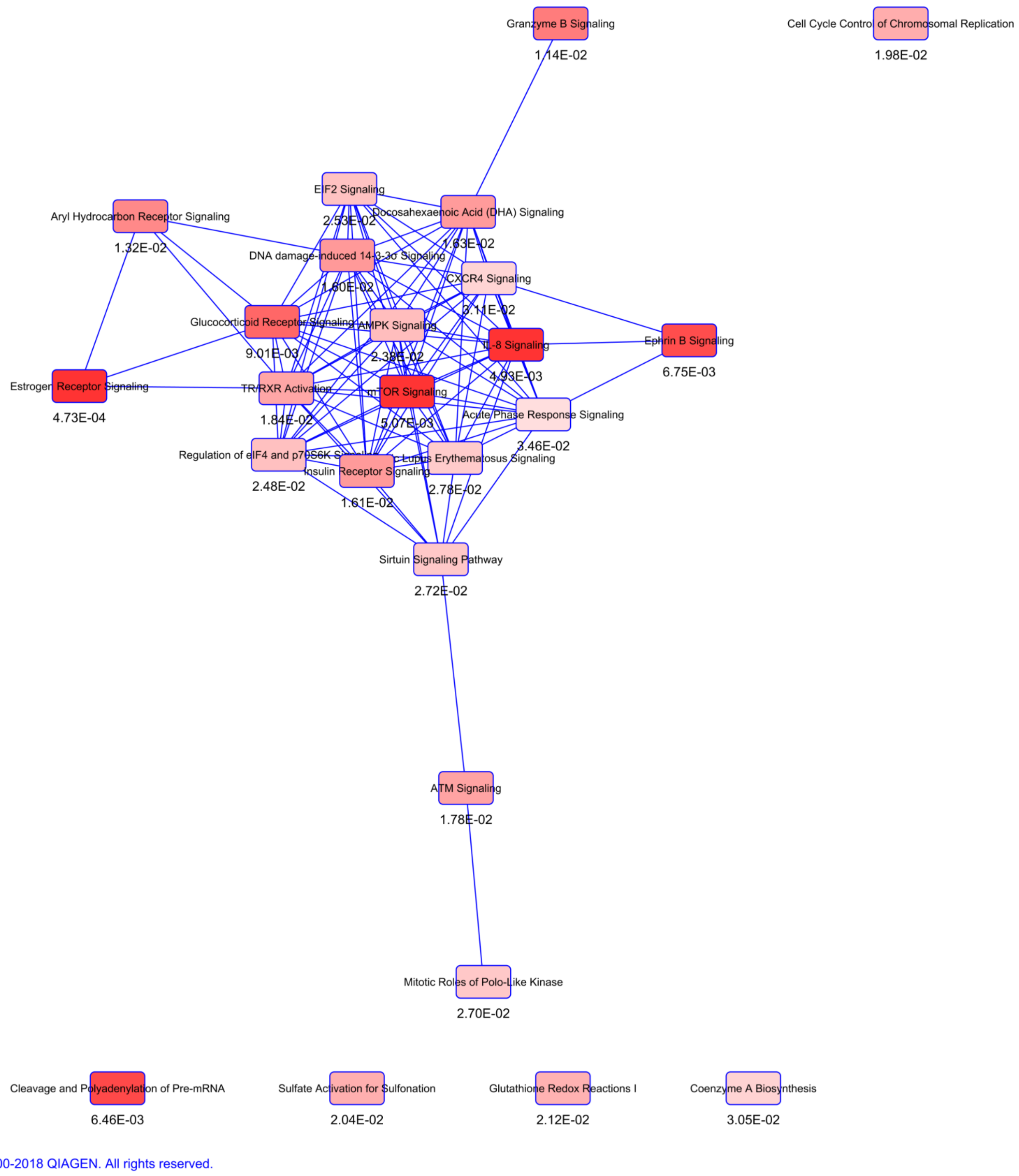

Figure S9. IPA Canonical Pathway Representation: Protein identifications that were unique to or increased by PROTAC treatment were analyzed by IPA (Qiagen) pathway analysis. Overlapping pathways from the dataset are displayed - increasing color indicates improved *p*-value (*p*-values are listed below each pathway).

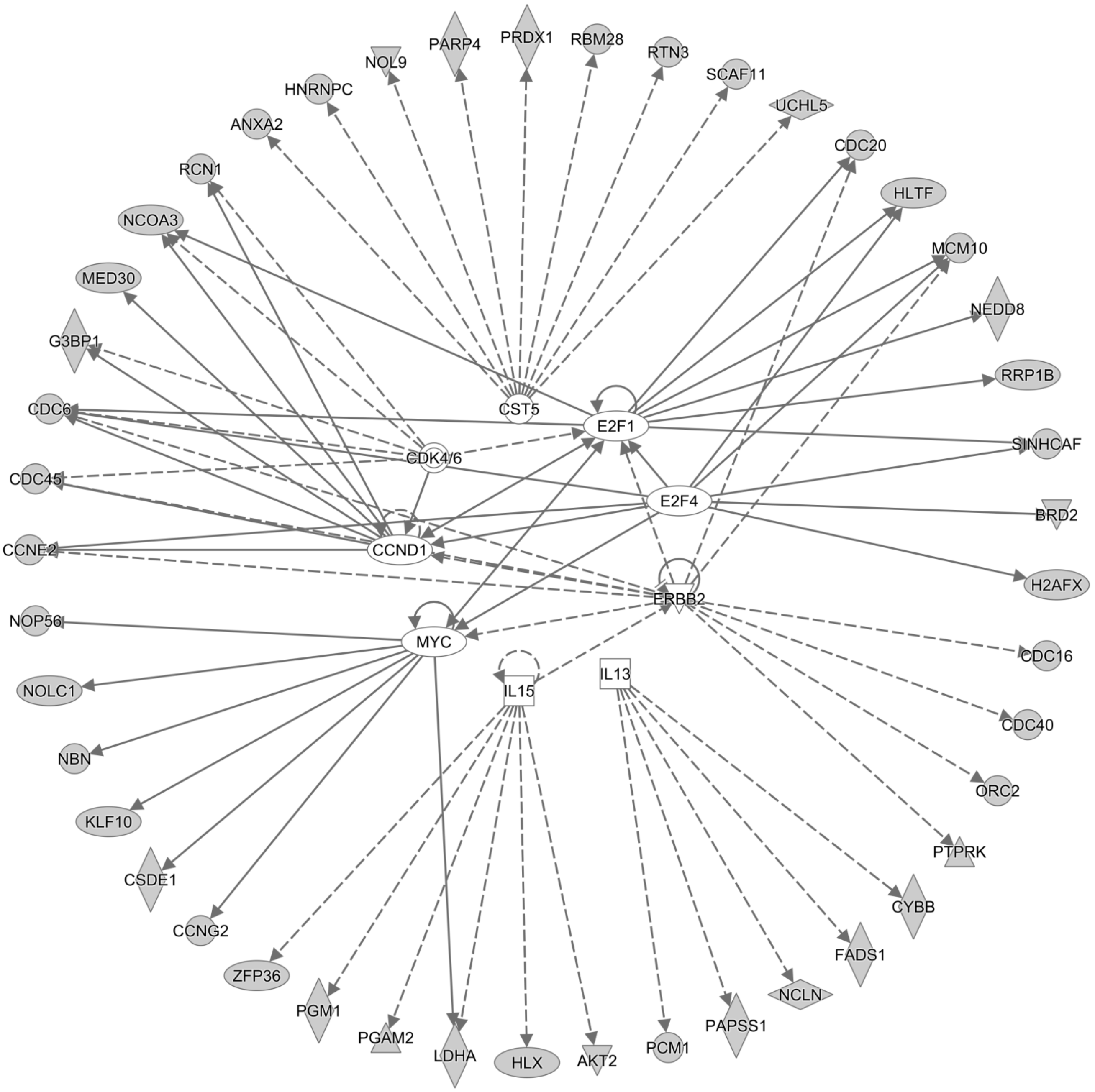

© 2000-2018 QIAGEN. All rights reserved.

Figure S10. IPA Upstream Regulator Prediction: Protein identifications that were unique to or increased by PROTAC treatment were analyzed by IPA (Qiagen) pathway analysis. Upstream regulators (white) of observed proteins/genes (gray) are depicted, the *p*-values determined are the following: ERBB2, 2.83E-03; CST5, 2.08E-03; E2F1, 7.82E-04; MYC, 1.29E-02; CCND1, 6.44E-03; E2F4, 1.16E-03; IL15, 7.04E-04; IL13, 3.81E-02; CDK4/6, 6.32E-04.
